# A Meta-Analysis on Randomised Controlled Clinical Trials Evaluating the Effect of the Dietary Supplement Chitosan on Weight Loss, Lipid Parameters and Blood Pressure

**DOI:** 10.3390/medicina54060109

**Published:** 2018-12-12

**Authors:** Cristina Moraru, Manuela Maria Mincea, Mirela Frandes, Bogdan Timar, Vasile Ostafe

**Affiliations:** 1 Department of Biology-Chemistry, Advanced Environmental Research Laboratory, West University of Timisoara, Timisoara 300086, Romania; cristina.g.moraru@gmail.com (C.M.); manuelamincea@yahoo.com (M.M.M.); 2 Department of Biostatistics and Medical Informatics, “Victor Babes” University of Medicine and Pharmacy, Timisoara 300041, Romania; mirela.frandes@umft.ro (M.F.); timar.bogdan@umft.ro (B.T.)

**Keywords:** chitosan, meta-analysis, body weight, overweight and obese patients, serum lipids, blood pressure

## Abstract

*Background and objectives*: Erratic results have been published concerning the influence of the dietary supplement chitosan used as a complementary remedy to decrease the body weight of overweight and obese people. The published articles mention as secondary possible benefits of usage of chitosan the improvement of blood pressure and serum lipids status. We performed a meta-analysis evaluating body weight, body mass index, total cholesterol, high density lipoprotein cholesterol, low density lipoprotein cholesterol, triglycerides, systolic and diastolic blood pressure among overweight and obese patients. *Materials and Methods*: Searching MEDLINE, Cochrane up to December 2017 on clinical trials that have assessed the influence of chitosan used as a dietary supplement on overweight and obese patients. An additional study was identified in the References section of another meta-analysis. A total of 14 randomised control trials (RCT) were used to assess the effect on body weight, serum lipids and blood pressure. *Results*: The usage of chitosan as a dietary supplement up to 52 weeks seems to slightly reduce the body weight (−1.01 kg, 95% CI: −1.67 to −0.34). Considering the other parameters studied, the most significant improvement was observed in systolic and diastolic blood pressure: −2.68 mm Hg (95% CI: −4.19 to −1.18) and −2.14 mm Hg (95% CI: −4.14 to −0.14) in favour of chitosan versus a placebo. *Conclusions*: Based on the meta-analysis realized with 14 RCT we concluded that the usage of chitosan as a dietary supplement can lead to a slight short- and medium-term effect on weight loss and to the improvement of serum lipid profile and cardiovascular factors.

## 1. Introduction

Overweight and obesity are predictors of health status being tightly correlated with alterations of blood pressure [1,2], blood lipids [3] and glucose homeostasis [4,5] as well as the presence of several cardiovascular diseases [6]. Overweight and obesity are defined by a body mass index (BMI) of 25–29.9 and ≥30 kg/m^2^, respectively [6] and are increasing rapidly in most of the European Union’s (EU) population. It was estimated that in 2014, 51.6% of the adult (18+) population of the EU was overweight [7], while the prevalence of obesity in women was 6.2–36.5% and in men it ranged from 4–28.3% [8,9]. The EU and USA have developed special programs aiming to reduce the burden of obesity and their related health complications, like the increased incidence of cardiovascular diseases, hypertension, stroke, type 2 diabetes, dyslipidaemia, musculoskeletal disorders and even some types of cancer and increased mortality [10,11,12,13,14,15].

Lifestyle optimisation is the first line of treatment for most patients who are overweight or obese. There is evidence suggesting that certain dietary supplements may be useful in the control and treatment of metabolic syndrome components like obesity, hypertension or lipid disorders [16,17].

**Figure 1:**
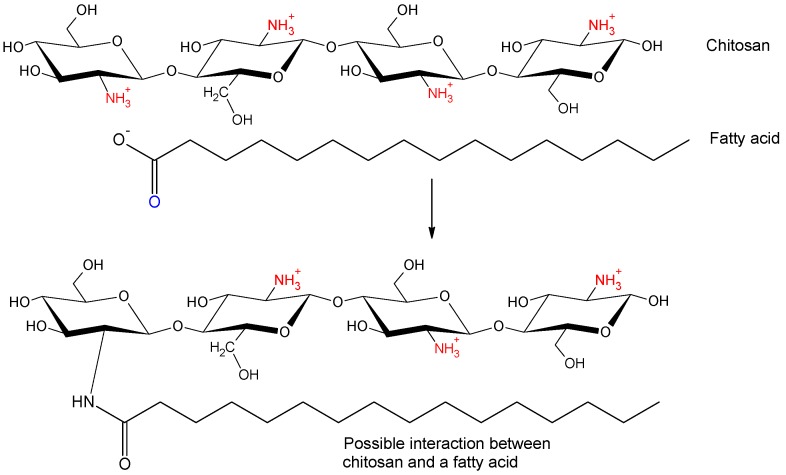
Possible interaction between positively charged chitosan in an acidic environment and a fatty acid [28].

Chitosan-a natural polysaccharide of β-1,4-linked glucosamine residues - is derived from chitin, the second most abundant biopolymer on the planet [18]. Chitosan results from the deacetylation of chitin [19,20]. It has been advocated as a weight loss supplement and adjuvant treatment for lowering blood lipids, glucose [21,22] and blood pressure [23,24,25,26]. The European Food Safety Authority (EFSA) Panel on Dietetic Products, Nutrition and Allergies (NDA) recommended a maximum intake of 3 g of chitosan per day [27]. In the acid aqueous medium of the stomach a positively charged gel forms as chitosan swells. The positive charge is due to the tertiary amino groups (−N^+^H_3_), formed from the amino groups of chitosan (−NH_2_, one per residue), which take on hydrogen ions (H^+^). These positively charged chitosan molecules attach strongly to the negatively charged fatty and bile acids [22,28]. Its cholesterol-lowering properties are due to the hydrophobic bonds it forms with cholesterol and other sterols, interfering with the emulsification process. The large polymer compounds resulting from this electrostatic and hydrophobic bonding are then weakly digested [28]. Figure 1 shows a possible interaction between chitosan and a fatty acid, according to a hypothesis advanced by Ylitalo [28].

Various clinical trials have been performed in the past 25 years investigating the effect of chitosan on body weight, body mass index (BMI), lipid parameters, blood pressure, glucose levels and other health characteristics [6,29,30,31,32,33]. The last meta-analysis quantifying these studies (the Cochrane systematic review of Jull 2008) concluded that these effects were probably not of clinical significance [34]. Additional clinical trials have been conducted since 2008, some of them with a higher number of participants and a longer duration.

Therefore, the present meta-analysis aims at comprehensively summarizing and quantifying the effects of short-term and medium-term chitosan supplementation as a weight loss treatment, in individuals with a body mass index ≥23.6, in RCTs.

## 2. Materials and Methods

### 2.1. Search Strategy

This study was designed according to the guidelines of the 2009 preferred reporting items for systematic reviews and meta-analysis (PRISMA) statement [35]. Medline (via Pubmed), EMBASE databases and the Cochrane Library were searched to identify randomised control trials (RCTs) examining the effects of chitosan supplementation on overweight and obese patients using the following search terms in titles and abstracts (also in combination with MESH terms): (“Chitosan”) and (“randomized controlled trial” or “obesity” or “overweight”). Only articles available in English were included. The search was limited to studies on humans. We also reviewed bibliographies of original research and previous reviews to complement the search. The literature was searched from inception to 31 December 2017. We also searched trial registries of ongoing trials. Additionally, we contacted authors to obtain additional data.

### 2.2. Study Selection

Original studies were included if they met the following inclusion criteria: (1) RCT (≥28 days follow-up) performed in subjects over 18 years of age; (2) individuals with a body mass index ≥23.6; (3) compared chitosan with placebo, (4) investigated the impact of chitosan on body weight and/or BMI, (5) used random allocation to the comparison groups and (6) presentation of sufficient information on baseline and at the end of the study in both chitosan and control groups. Additional parameters like blood lipids: plasma total cholesterol (TC), high density lipoprotein cholesterol (HDL-C), low density lipoprotein cholesterol (LDL-C), and triglycerides (TG); systolic blood pressure (SBP) and diastolic blood pressure (DBP) were also included if available.

Exclusion criteria were: (1) non-clinical studies; (2) uncontrolled trials; (3) studies reported in abstract form or letters only and (4) lack of sufficient information on baseline or follow-up parameters. Exclusion of an article for the latter reason was applied if no feedback was received after contacting the author(s).

### 2.3. Data Extraction

Eligible studies were reviewed and from each study the following data were abstracted: first author’s name; year of publication; study location; study design (crossover or parallel; single blind or double blind); intervention duration; form and dosage of chitosan intake; number of participants in the chitosan and control groups; age, gender and body mass index (BMI) of study participants; circulating concentrations of total cholesterol, LDL-C, HDL-C and triglycerides; systolic and diastolic blood pressure.

The data was extracted by two reviewers using a four-eye principle. The data included general trial characteristics, baseline parameters of individuals and outcome parameters (weight, BMI, cholesterol, triglycerides and blood pressure). Disagreement was resolved by consensus.

### 2.4. Quantitative Data Synthesis and Analysis

Meta-analysis techniques were used to combine the results from distal follow-up (the last follow-up reported) from independent studies [36]. Effect size was expressed as the weighted mean difference (WMD) and 95% confidence intervals (CI) between chitosan supplementation and placebo for continuous outcomes. Heterogeneity across the studies was tested using the I^2^ index.

A random-effects model and the generic inverse variance method were used to compensate for the heterogeneity of studies in terms of study design, chitosan dose and demographic characteristics (e.g., age, gender, underlying disease and comorbidities) of populations studied.

All tests were two-tailed and a *p*-value of <0.05 was considered statistically significant in all analyses. The pooled estimates of the effect were calculated using Review Manager (RevMan) (Computer program, Version 5.3. Denmark, Copenhagen: The Nordic Cochrane Centre, The Cochrane Collaboration, 2014). Plasma lipids (TC, LDL-C, HDL-C and TG) concentrations were expressed in mmol/L. Dividing by 38.6 and 88.5 was used to convert cholesterol (TC, LDL-C, HDL-C) and TG, respectively, expressed in mg/dL into mmol/L.

## 3. Results

### 3.1. Retrieved Data and Characteristics of the Trials

**Figure 2:**
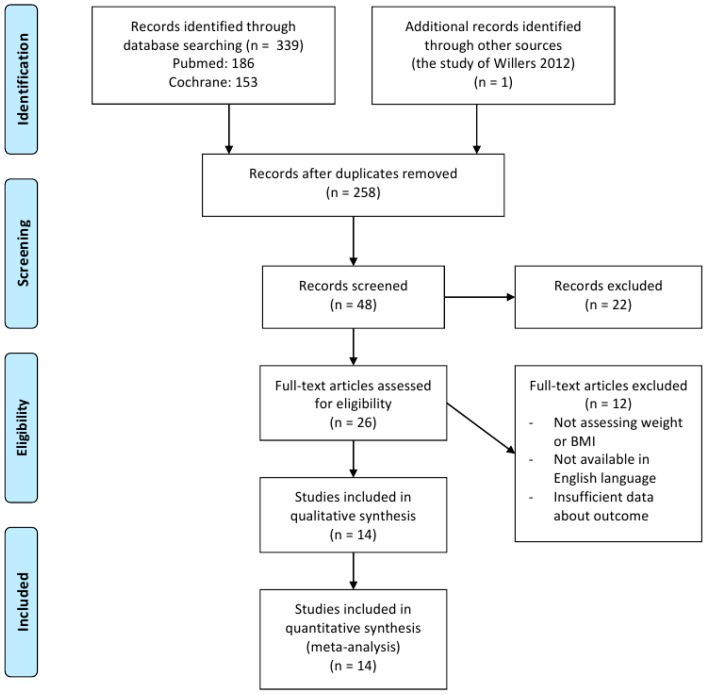
Flowchart of the clinical trial selection process showing the number of studies at each selection step.

Twenty-six full text articles were assessed for eligibility, 12 studies were excluded for not assessing or not providing data about weight or BMI. Figure 2 shows the number of studies assessed and excluded through the stages of the meta-analysis.

A summary of the basic characteristics of the included trials and participants is given in Table 1. After final assessment, 14 RCTs fulfilled the inclusion criteria and were preferred for the final meta-analysis.

In total, 1101 participants were randomised, of whom 570 were allocated to the chitosan and 531 allocated to the placebo. The mean trial duration was 17 weeks (range 4–52 weeks) and mean study size was 79 participants (range 12–250). The studies were conducted in New Zealand [37], the United Kingdom [23], the United States [38,39], Canada [31,40], Italy [29,30], Japan [26], Germany [6,30], Mexico [32], Singapore [41] and Spain [33].

Most studies included men as well as women (except for Schiller 2001 [38] and Bokura 2003 [26], women only). Ho 2001 [41] presented separate results for men and women. The age of the study participants was from 18–70 years. The criteria for overweight and obese varied from study to study. Chitosan dosage ranged from 0.34 g/day–3.4 g/day (mean 2 g/day) and one study [40] did not report any dose. Preparations containing other active substances were used in five studies [29,33,38,39,40] while other studies used only chitosan. Table 1 presents these characteristics.

The efficacy of chitosan at various doses coupled with calorie restriction was investigated [30] Other studies advised their patients not to modify their habitual diet or lifestyle [23,26,29,31,32,33,38,40]. Two studies [23,41] did not report significant body weight reduction in the chitosan group compared to the placebo group, while the others found that chitosan led to significant weight loss.

### 3.2. Types of Outcome Measures

The studies included in this meta-analysis did not perform any extended follow-up beyond the intervention period, i.e., the outcomes were measured post-intervention only.

#### 3.2.1. Body Weight

**Figure 3:**
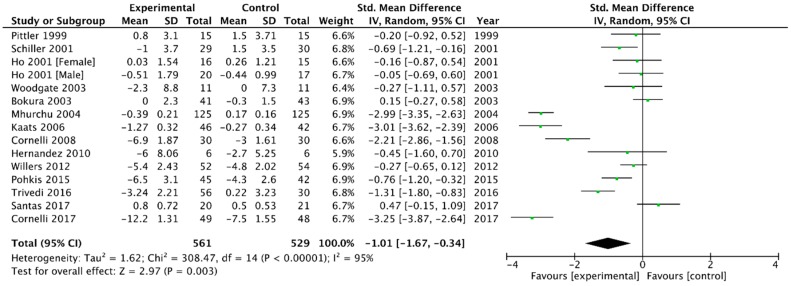
Forest plot depicting the effect of chitosan on body weight (kg) using a random-effect model.

Combining the 14 trials that provided data on body weight [6,23,26,29,30,31,32,33,37,38,39,40,41,42], application of a random-effect model produced a weighted mean difference (WMD) in body weight of −1.01 kg (95% CI, −1.67 to −0.34) in favour of chitosan versus placebo (*p* = 0.03, I^2^ = 95%) (Figure 3).

#### 3.2.2. Body Mass Index (BMI)

**Figure 4:**
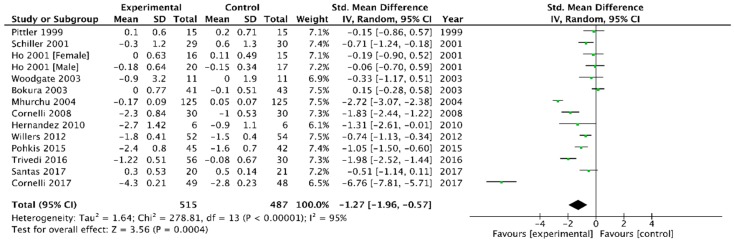
Forest plot depicting the effect of chitosan on body mass index (kg/m^2^) using a random–effect model.

Combining the 13 trials that provided data on BMI [6,23,26,29,30,31,32,33,37,38,40,41,42] using the application of a random-effect model produced a WMD in BMI of −1.27 kg/m^2^ (95% CI −1.96 to −0.57) in favour of chitosan versus placebo (*p* = 0.0004, I^2^ = 95%) (Figure 4).

#### 3.2.3. Blood Pressure

##### Systolic Blood Pressure

**Figure 5:**
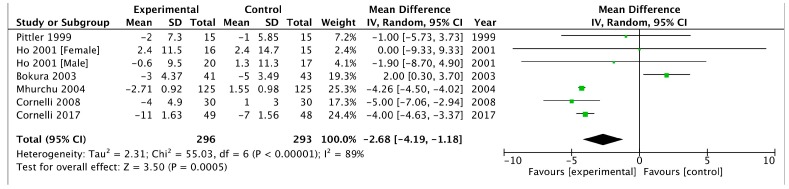
Forest plot depicting the effect of chitosan on systolic blood pressure (mm Hg) using a random-effect model.

Combining the six trials that provided data on SBP [23,26,29,37,41,42] using a random-effect model produced a WMD in SBP of −2.68 mm Hg (95% CI, −4.19 to −1.18) in favour of chitosan versus the placebo (*p* = 0.0005, I^2^ = 89%) (Figure 5).

##### Diastolic Blood Pressure

**Figure 6:**
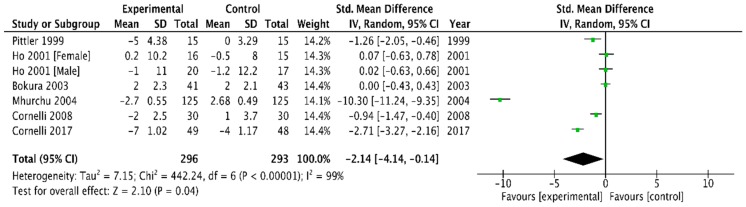
Forest plot depicting the effect of chitosan on diastolic blood pressure (mm Hg) using a random-effect model.

Combining the six trials that provided data on DBP [23,26,29,37,41,42], the application of a random-effect model produced a WMD in DBP of −2.14 mm Hg (95% CI, −4.14 to −0.14) in favour of chitosan versus the placebo (*p* =0.04, I^2^ = 99%) (Figure 6).

Changes in SBP: mean difference: −2.68 mm Hg (−4.19 to −1.18), *p* = 0.0005 and DBP: mean difference: −2.14 mm Hg (−4.14 to −0.14), *p* = 0.04 was significantly different between chitosan and placebo. Heterogeneity was high for both systolic and diastolic blood pressure (Figure 5 and Figure 6).

#### 3.2.4. Plasma Cholesterol and Triglyceride Concentrations

##### Total Cholesterol

**Figure 7:**
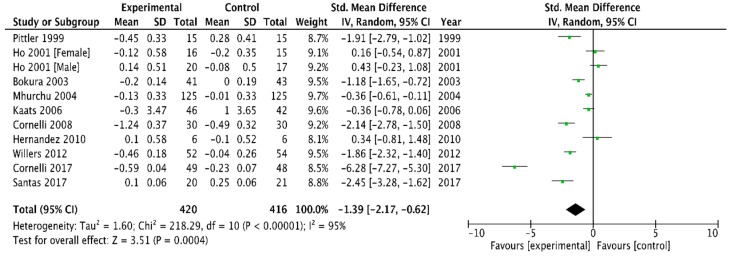
Forest plot depicting the effect of chitosan on total cholesterol (mmol/L) using a random-effect model.

Combining the 10 trials that provided data on total cholesterol [6,23,26,29,32,33,37,39,41,42] using a random-effect model produced a WMD in total cholesterol of −1.39 mmol/L (95% CI, −2.17 to −0.62) in favour of chitosan versus a placebo (*p* = 0.0004, I^2^ = 95%). Heterogeneity was high (Figure 7).

##### HDL Cholesterol

**Figure 8:**
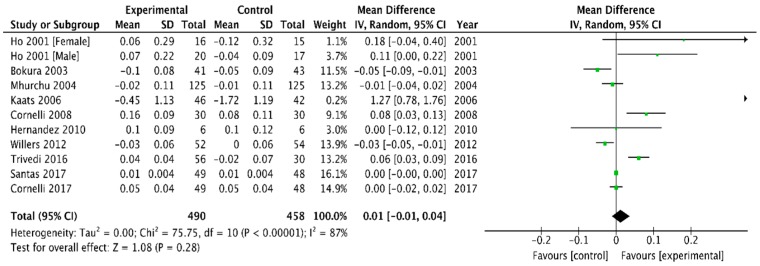
Forest plot depicting the effect of chitosan on high density lipoprotein cholesterol (HDL) (mmol/L) using a random-effect model.

The mean changes in HDL cholesterol concentration were available for 10 studies [6,26,29,31,32,33,37,39,41,42]. Concentration increased more after chitosan than after the placebo treatment (0.01 mmol/L (95% CI, −0.01 to 0.04), *p* = 0.28). Heterogeneity was high, I^2^ = 87% (Figure 8).

##### LDL Cholesterol

**Figure 9:**
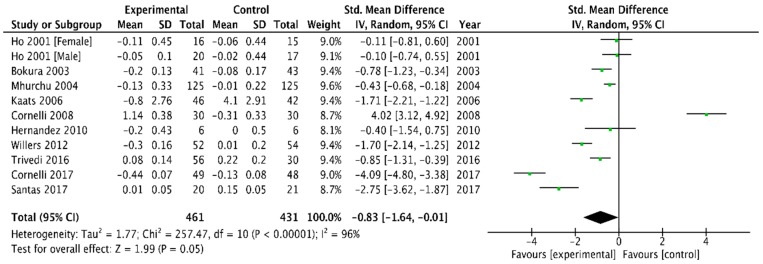
Forest plot depicting the effect of chitosan on low density lipoprotein cholesterol (LDL) (mmol/L) using a random-effect model.

The mean changes in LDL cholesterol concentration were available for 11 studies [6,26,29,31,32,33,37,39,41,42]. Concentration decreased after chitosan supplementation compared with placebo group: (−0.83 mmol/L, 95% CI, −1.64 to −0.01, *p* = 0.05). Heterogeneity was high, I^2^ = 96% (Figure 9).

##### Triglycerides

**Figure 10:**
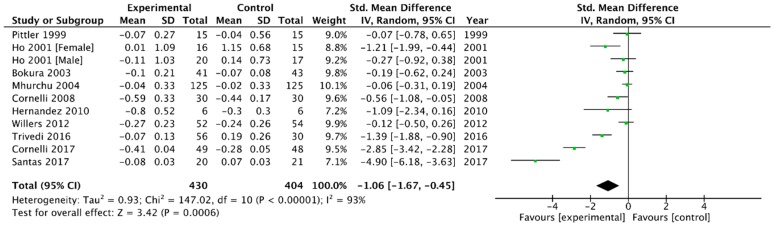
Forest plot depicting the effect of chitosan on triglycerides (mmol/L) using a random-effect model.

Combining the 10 trials that provided data on triglycerides levels [6,23,26,29,31,32,33,37,41,42] using a random-effect model produced a WMD in triglycerides of −1.06 mmol/L (95% CI, −1.67 to −0.45) in favour of chitosan versus placebo (*p* = 0.0006, I^2^ = 93%) (Figure 10).

## 4. Discussion

Chitosan has been studied as an agent for lowering weight, cholesterol and blood pressure since it is not well digested in the human body. Chitosan appears to bond with fatty compounds in the digestive tract, carrying them out in the faeces [34].

Chitosan and its derivatives have been widely promoted and are freely available in health stores and pharmacies [24]. In 2015 the global market for chitosan valued 3.19 billion USD, Japan covering 35% of chitosan production [43].

A number of human trials have reached different conclusions concerning the effect of chitosan on weight loss. Although early studies conducted with hypocaloric diets suggested that chitosan had a significant effect on body weight [44,45,46], later clinical trials showed a much smaller effect [38], or no effect at all when compared to a placebo [23,37]. The meta-analysis of Ernst [47] suggested a 3.3 kg greater weight loss with chitosan compared with placebo, while two more recent meta-analyses [34,48] suggested only a 1.7 kg weight loss.

Since then, the efficacy of chitosan was investigated at different doses and also when administered concomitantly with a calorie-restricted diet and physical activity in various short-term and medium-term studies.

This meta-analysis provides an overview on the efficacy of chitosan as a treatment for overweight and obesity. The authors compared the effect of chitosan and placebo for overweight and obese patients on factors such as weight, BMI and other parameters related to metabolic disease. The overall finding of this meta-analysis is that chitosan may be effective as an adjuvant treatment in weight loss programs.

In the studies analysed in this meta-analysis, the daily intake of chitosan varied from 0.34–3.4 g/day. One study did not mention the amount of chitosan administered to the patients [40].

### 4.1. Comparison with Other Meta-Analysis

Our results are supported by two systematic reviews that showed some weight loss and further improvements in cardiovascular risk factors after chitosan supplementation compared with a placebo treatment [34,48]. Both meta-analyses showed a mean weight loss of 1.7 kg.

The systematic review of Ni Mhurchu [48] included 14 randomised placebo-controlled clinical trials with a total of 1131 participants assessing chitosan as a weight reduction agent. The duration of the studies ranged from 4–24 weeks. The results showed a slight reduction of weight compared with the placebo.

The meta-analysis of Jull [34] that compared and reviewed 15 randomised controlled trials, reported evidence that chitosan was more effective than a placebo in the treatment of overweight and obesity, high blood pressure and slightly effective for lowering total cholesterol and triglycerides. The study included 1219 patients with treatment duration of 4–24 weeks.

Both previous systematic reviews mentioned that the results of several of the included trials should be interpreted with caution due to their poor quality and unsuccessful correspondence with their authors [34,48]. Overall results from high quality trials only demonstrated minimal effect from chitosan on body weight.

The present meta-analysis reports that chitosan oral supplementation led to greater body weight loss than placebo treatment for obesity for up to 52 weeks of follow–up. The mean weight difference of −1.01 kg achieved over trial periods ranging from 4–52 weeks was minor. However, our analysis showed a significantly greater decrease in intervention group in comparison with the placebo for TC, LDL cholesterol, and TG. Furthermore, HDL cholesterol increased after chitosan supplementation. Decreasing TC, LDL, TG and increasing HDL represents an important clinical target [49]. The effect of chitosan on total cholesterol levels was similarly reduced (−1.39 mmol/L (95% CI, −2.17 to −0.62)) but remained statistically significant (P < 0.0004) although its clinical significance is also questionable.

In our meta-analysis on overweight and obese patients the chitosan supplementation was associated with a significant decrease in SBP and DBP with −2.68 mm Hg (95% CI −4.19 to −1.18) and −2.14 mm Hg (−4.14 to −0.14) in favour of chitosan versus placebo. The Heart Outcome Prevention study showed that a decrease in SBP of 2–3 mm Hg in patients with diabetes and one other risk of cardiovascular morbidity was associated with a 25% reduction in risk of myocardial infarction, or cardiovascular death [50].

Table 2 presents a comparison between the meta-analysis of Jull 2008 [34] and our meta–analysis. The systematic study of Jull [34] showed a slight improvement in cardiovascular risk factors, while our meta-analysis showed more improvement on cholesterol, TG and BMI.

The most common adverse events after chitosan treatment in analysed studies were constipation and diarrhoea.

### 4.2. Strengths and Limitations of Study

The interpretation of these meta-analysis results should take into account that several studies also used other active substances in addition to chitosan, were of limited size or duration (are 3 months or less) or did not include parameters like blood pressure, cholesterol, and triglycerides. In addition, some potentially informative trials were not included if the participants’ weight or BMI was not assessed [51,52,53].

Our study did not include five clinical trials published in 1995–1996 [44,45,46,54,55] since correspondence with their authors was unsuccessful. Concerns were expressed about a systematic bias in these studies since the chitosan was supplied by one manufacturer and the studies appeared in the same Italian journal [34,47]. A meta-analysis study based on these trials reported a significant effect of chitosan as a weight loss supplement (−3.3 kg) [47], however later studies reported that the difference in terms of weight loss between control and chitosan was considerably smaller (−1.7 kg) [34,48].

## 5. Conclusions

This meta-analysis of data from 14 RCT provided comprehensive evidence that compared with the placebo, chitosan supplementation leads to a slight short- and medium-term effect on weight loss of chitosan supplementation, improves plasma lipid profile and cardiovascular outcomes. In summary, this meta-analysis establishes a beneficial effectiveness on overweight and obese patients. Considering the impact of the aforementioned factors on the overall health state of the patients, we may conclude that chitosan intervention improves the overall prognosis of these people. However, additional long-term research is needed, stating all of the characteristics of the chitosan formulations.

## Figures and Tables

**Table 1:** Characteristics of the included studies.

No.crt.	Reference (Author, Year)	No. of Participants	Study Design **	Participants’ Age (Years)	Participants’ Gender	Participants’ Baseline BMI (kg/m^2^)	Chitosan Dosage Form	Chitosan Administration (g/Day)	Duration (Days)	Confounding Variables (Other Interventions or Active Ingredients)
1	Pittler, 1999	30	R, DB	18–60	M, F	treatment: 26.3 placebo: 26.9	capsules	1	28	Participants were excluded if they were currently following a diet.
2	Schiller, 2001	59	R, DB	21–55	F	treatment: 32.2 placebo: 31.8	capsules	3	56	Subjects were instructed to continue their regular caloric intake. The chitosan capsules contained >90% chitosan and <10% succinic acid.
3	Ho 2001 (Male)	37	R, DB	treatment: 42.4 * placebo: 42.5 *	M	treatment: 25.7 placebo: 27.0	capsules	3.1	84	No restrictions or monitoring of dietary habits.
	Ho 2001 (Female)	31	R, DB	treatment: 42.8 * placebo: 44.3 *	F	treatment: 25.6 placebo: 24.6	capsules	3.1	84	No restrictions or monitoring of dietary habits.
4	Bokura 2003	84	R, DB	34–70	F	treatment: 23.6 placebo: 22.3	capsules	1.2	56	Subjects were instructed to continue their regular diet.
5	Woodgate, 2003	22	R, DB	20–50	M, F	treatment: 36.8 placebo: 34.6	capsules	no information available	42	Subjects were instructed to continue their regular diet and exercise patterns. The capsules contained additional active ingredients (glucomannan, chitosan, fenugreek, G sylvestre, and vitamin C)
6	Mhurchu, 2004	250	R, DB	>18	M, F	treatment: 34.8 placebo: 36	capsules	3	168	All participants received standardized dietary and lifestyle advice for weight loss.
7	Kaats 2006	88	R, DB	treatment: 43.9 * placebo: 48.7 *	M, F	not available not available	capsules	3	60	Subjects were asked to follow a behavior modification plan and their physical activity was monitored. The treatment group took the following substances in addition: Beta-glucan, sno white oat fiber, betaine hydrochloride and aloe saponins (1 mg of each).
8	Cornelli, 2008	56	R, DB	30–60	M, F	treatment: 27.4 placebo: 27.4	tablets	2	122	Intake of at least 1.5 L of water per day. Patients were asked to keep their habitual diet. In addition to chitosan, the capsules contained L-ascorbic acid (6%) and tartaric acid (3%).
9	Hernandez, 2010	12	R, DB	30–50	M, F	treatment: 34.3 placebo: 32.7	no information available	2.25	91	All patients received general recommendations about their medical nutritional therapy and were instructed to not modify their usual forms of exercise.
10	Willers, 2012	120	R, DB	30–60	M, F	treatment: 31.7 placebo: 31.7	tablets	0.8	84	One serving of protein-rich formula diet a day.
11	Pohkis 2015	87	R, DB	21–75	M, F	treatment: 35 placebo: 35	tablets	3.4	175	A daily calorie deficit (500 cal) and an increased daily physical activity (7 MET ***-h/ week).
12	Trivedi, 2016	96	R, SB	18–65	M, F	treatment: 30.93 placebo: 30.91	capsules	2.5	90	Subjects were advised to maintain their normal routine diet.
13	Cornelli, 2017	97	R, DB	25–65	M, F	treatment: 33.9 placebo: 34.1	tablets	1.6	365	10% calorie restriction and an increase in physical activity (9 MET-h/wk).
14	Santas, 2017	41	R, DB	18–65	M, F	treatment: 29.1 placebo: 29.2	caps with solvable content	0.343	84	Diet was not controlled and participants were asked not to alter their dietary habits and physical activity. The administered caps contained a beta-glucan-chitin-chitosan fraction (BGCC)

* Authors provided only the participants’ mean age. BMI-body mass index. ** R: Randomised; DB: double-blind; SB: single-blind. *** MET: Metabolic Equivalent of Task.

**Table 2:** Comparison between the results obtained in our meta-analysis and a previous meta-analysis.

Parameter	Jull [35]	Our Study
Weight	−1.71 (−2.09, −1.32)	−1.01 (−1.67, −0.34)
BMI	−0.35 (−0.55, −0.15)	−1.27 (−1.96, −0.57)
TC	−0.21 (−0.28, −0.13)	−1.39 (−2.17, −0.62)
HDL	0.03 (0.01, 0.05)	0.01 (−0.01, 0.04)
LDL	−0.16 (−0.23, −0.10)	−0.83 (−1.64, −0.01)
TG	−0.12 (−0.19, −0.06)	−1.06 (−1.67, −0.45)
SBP	−5.94 (−7.25, −4.63)	−2.68 (−4.19, −1.18)
DBP	−3.38 (−4.35, −2.42)	−2.14 (−4.14, −0.14)
